# The Prairie Vole Model of Pair-Bonding and Its Sensitivity to Addictive Substances

**DOI:** 10.3389/fpsyg.2019.02477

**Published:** 2019-11-06

**Authors:** Sheena Potretzke, Andrey E. Ryabinin

**Affiliations:** Department of Behavioral Neuroscience, School of Medicine, Oregon Health & Science University, Portland, OR, United States

**Keywords:** mating, pair-bond, partner preference, prairie vole, oxytocin, corticotropin releasing factor, alcohol

## Abstract

The prairie vole (*Microtus ochrogaster*) is an extensively studied model for understanding the neural mechanisms underlying social affiliations and pair bonds. With clearly observed face and construct validity, this species offers translational insights into mechanisms involved in intimate relationships in humans. Moreover, the prairie vole model promises to advance our understanding – as well as allow for predictions – of the effects of extraneous factors (not normally encountered in nature) on such relationships. This mini review describes some of the neurobiological mechanisms regulating social affiliation in prairie voles, followed by an overview of the effects of alcohol and other drugs of abuse on formation and maintenance of pair-bonds. Based on available literature, we demonstrate that the effects of such extraneous factors on formation and maintenance of pair-bonds are sex-dependent, as well as dependent on the specific nature of the addictive drug. In turn, the lack of similarities in effects of different addictive substances on pair-bond formation suggests that these substances engage different neurocircuits that may or may not overlap with neurocircuits involved in various social behaviors. This lack of consistency of effects across studied drugs of abuse indicates the need to further examine the effects of individual drugs on affiliative behaviors. We highlight the deficiencies in this field of research, particularly the sparsity of studies on effects of drugs of abuse on the maintenance of established bonds. Future investigations in this field could help design strategies to help afflicted individuals.

## Introduction

Reproduction, whether asexual or sexual, is of the upmost importance to the survival of a species. Consequently, organisms have evolved various mating systems to ensure reproduction. Nevertheless, throughout the animal kingdom, promiscuity reigns supreme. Approximately 95–97% of mammals utilize this mating strategy, while the remaining 3–5% exhibit social monogamy (Lukas and Clutton-Brock, 2013; Johnson and Young, 2015). Because social monogamy does not require sexual exclusivity, this strategy can provide a valuable insight into biological aspects of social attachments.

Research on the prairie vole (*Microtus ochrogaster*) – a socially monogamous rodent species – allowed for characterization of the neurobiological underpinnings of the pair-bond. Moreover, the effects of alternative rewards and addictive substances on pair-bonds can be investigated by utilizing these animals. This review focuses on the prairie vole model of pair-bonding, its translational value to human social attachments, and its sensitivity to the effects of alcohol and drugs of abuse.

## Pair Bond as the Hallmark of Social Monogamy

Pair-bonds are commonly described as enduring, preferential associations between two sexually mature adults, characterized by selective affiliation, contact, and mating with the partner over a stranger, which is generally called partner preference (PP; Young et al., 2011). Pair-bonded animals also show aggression toward sexual competitors – called “mate-guarding” – and biparental care of offspring (Kleiman, 1977; Buss, 1988; Fraley et al., 2005). These are social behaviors also seen in humans. The occurrence of sociosexual attachments in nearly all human civilizations provides compelling evidence in support of these attachments being intrinsic to human social behavior (Young et al., 2011).

There are physiological and psychological advantages of pair-bonds in humans. Paired individuals live longer than unpaired individuals across all demographic groups (House et al., 1988; Lillard and Waite, 1995). Interestingly, the level of intimacy between two bonded individuals is positively correlated with immune function and cardiovascular health, while it is inversely correlated with depressed mood (Millard et al., 1988; Kiecolt-Glaser and Newton, 2001). Importantly, socially monogamous behaviors appear to be facilitated by distinct and evolutionary conserved neural mechanisms that mediate selective social attachments.

## Neurobiology of Pair Bonding

Dopamine (DA) signaling is implicated in the formation, expression, and maintenance of pair-bonds. Prairie voles display higher densities of DA2 receptors (D2Rs) and decreased expression of DA1 receptors (D1Rs) in the medial prefrontal cortex (mPFC), as well as a lower density of D1Rs in the nucleus accumbens (NAcc), compared to promiscuous meadow voles (Aragona et al., 2006; Smeltzer et al., 2006). Mating increases DA activity and D1R:D2R signaling ratio in the NAcc, facilitating PP formation (Young et al., 2011; Resendez and Aragona, 2013). D2R activation is necessary and sufficient for PP formation in both male and female prairie voles (Gingrich et al., 2000; Aragona et al., 2006). Following formation, bond maintenance is ensured by increased D1R expression in the NAcc (Aragona et al., 2006; Resendez and Aragona, 2013). In addition, DA cells have been found in the bed nucleus of the stria terminalis (BNST) and the medial amygdala (MeA) in the prairie vole but not in the meadow vole (Northcutt et al., 2007). The larger implication of studies in diverse species such as zebra finch and coppery titi monkeys is support for an evolutionarily conserved contribution of these reward and learning pathways to pair-bonding (Bales et al., 2007; Banerjee et al., 2013). Indeed, recent imaging studies point to the associations between levels of D2/3Rs in the ventral striatum and self-reported social attachment (Caravaggio et al., 2017), and to increased DA activity in the MeA during bonding in humans (Atzil et al., 2017).

Oxytocin (Oxt) is a conserved nonapeptide mediating species-specific social and maternal behaviors (Pedersen and Prange, 1979; Ferris et al., 1984; Kendrick et al., 1987; Argiolas and Melis, 2005). The distribution of Oxt receptors (Oxtr) varies within and across species (Anacker and Beery, 2013; Albers, 2015). Specifically, socially monogamous voles display higher densities of Oxtr in the BNST, mPFC, and NAcc but lower levels of Oxtr binding in the ventromedial hypothalamus, LS, and anterior cortical amygdala (Insel and Shapiro, 1992; Young et al., 1996; Smeltzer et al., 2006). Oxtr expression within mesolimbic pathways is critical for pair-bonding (Young et al., 2011). Furthermore, the Oxt and DA systems interact in their functions related to pair-bonding (Liu and Wang, 2003). In humans, Oxt and Oxtr are also closely associated with social behaviors (Ebstein et al., 2009; Heinrichs et al., 2009; Meyer-Lindenberg et al., 2011). Perhaps most interestingly, Oxtr gene variants are associated with relationship status (Walum et al., 2008, 2012), and Oxt levels within blood plasma can predict success rates in romantic relationships (Schneiderman et al., 2012).

Arginine vasopressin (AVP), a peptide similar to Oxt, is also implicated in the regulation of social bonding. AVP receptor 1a (AVPR1a) expression is higher in the ventral pallidum (VP) and LS in monogamous versus promiscuous vole species (Nair and Young, 2006), and AVP signaling in VP and LS is causally linked to PP (Liu et al., 2001; Lim et al., 2004; Donaldson et al., 2010). On the other hand, mate-guarding in prairie voles is dependent on AVPR1a signaling in the anterior hypothalamus (Gobrogge et al., 2009). AVPR1a in the retrosplenial cortex is important for the regulation of monogamous behaviors in wild prairie voles (Okhovat et al., 2015; Ophir, 2017). In agreement with the translational value of these findings, AVPR1 polymorphisms are associated with effects of childhood adversity on social interactions in adulthood (Liu et al., 2015). Moreover, administration of AVP increased empathic concerns and risky cooperative behaviors in humans (Tabak et al., 2015; Brunnlieb et al., 2016).

Pair-bonding also involves the corticotropin releasing factor (CRF) receptor system. Monogamous voles display lesser levels of CRFR1 and greater levels of CRFR2 binding within the NAcc (Lim et al., 2005, 2006). Administration of CRF into either the cerebral ventricles or intra-NAcc promoted PP formation in male prairie voles, and effects are prevented by concurrent administration of either a CRFR1 or CRFR2 antagonist (DeVries et al., 2002; Lim et al., 2007). These effects involve either CRF or urocortin 1, since the latter peptide has higher affinity than CRF to these receptors. Indeed, urocortin 1 also shows higher levels of expression in the centrally projecting Edinger-Westphal nucleus (EWcp) in promiscuous versus monogamous vole species (Lim et al., 2005, 2006). The contributions of the CRF system to social attachment are translationally relevant as human polymorphisms in the CRHR1 gene (encoding CRFR1) moderate loneliness in older adults (Chou et al., 2014) and effects of early life stress on emotional empathy (Grimm et al., 2017). Thus, collective neuroplastic abilities of these evolutionarily conserved and connected systems are responsible for the formation and maintenance of the pair-bond.

## Effects of Addictive Substances on Social Bonding in Humans

Addictive substances profoundly affect human social behavior. Many addictive substances are taken in social circumstances and are often expected to promote social bonding. However, drug abuse is associated with deleterious effects on social relationships; in fact, alcohol and drug abuse are the third most cited reason for divorce in the United States (Amato and Previti, 2003). Because of the difficulties in obtaining data on the use of illicit drugs, researchers often combine data from several drugs to increase the statistical power. These studies consistently point to the negative association between drug abuse and social bonding, relationship stability, and relationship satisfaction (Dull, 1984; Fals-Stewart et al., 1999). This association is much better followed for addictive substances that are used legally, like alcohol.

While confirming the negative effect of heavy alcohol use on various measures of social bonding, research also identified differences between modes of alcohol drinking within couples. Specifically, couples in which only one spouse drinks heavily (discordant) are less stable than couples in which both spouses drink heavily (concordant) or abstinent couples, while concordant couples are significantly more stable than discordant drinking couples and may be just as stable as abstinent couples (Marshal, 2003; Ostermann et al., 2005; Torvik et al., 2013; Leonard et al., 2014). Additionally, rates of marital dissatisfaction and separation are higher among couples when there is a difference in alcohol consumption between partners (Mudar et al., 2001; Homish and Leonard, 2007; Homish et al., 2009). Interestingly, while this difference in rates of separation is observed in relation to alcohol, neither concordant nor discordant tobacco or marijuana use is associated with increased divorce (Leonard et al., 2014). The latter data indicate that while addictive substances have strong negative effects on the stability of human bonds, there are differences between specific drugs that should be examined. Intriguingly, while socioeconomic factors impact the stability of a marriage, these factors do not moderate effects of addictive substances on marital stability, suggesting involvement of biological factors (Ribar and Kenkel, 1994; Leonard et al., 2014).

## Prairie Voles as Model of Effects of Addictive Substances on Pair Bonding

While epidemiological research on associations between the use of specific drugs of abuse and social effects is being increasingly performed, assessing causal relations between factors requires the use of animal models. Traditional laboratory animals (i.e., mice and rats) are not very suitable for these experiments because they do not display social monogamy. By contrast, prairie voles offer a well-established model of pair bonding and affiliative behaviors. In addition, prairie voles freely prefer alcohol solutions over water (Anacker et al., 2011) and can also consume solutions of methamphetamine (Hostetler et al., 2016).

Early work investigating the influence of social factors on rewarding properties of drugs showed that pair-bond formation reduces amphetamine (AMPH) seeking as evaluated by conditioned place preference (CPP; Liu et al., 2010, 2011). CPP pairs a context with a stimulus, in this case a drug, and assesses preference for the paired context through comparison of time spent in the paired versus alternative, non-paired context. CPP does not assess effects of voluntary exposure to a drug and is accompanied by stress of drug administration. Therefore, subsequent studies used voluntary modes of self-administration, focusing on alcohol consumption. These studies demonstrated existence of social facilitation and social inhibition of alcohol drinking, as well as effects of social hierarchies on alcohol drinking (Anacker et al., 2011, 2014b; Hostetler et al., 2012; Hostetler and Ryabinin, 2014) – both increasing and decreasing alcohol consumption dependent on a number of contextual variables. These first experiments were performed in same-sex pairs of prairie voles. More recent studies observed facilitation of alcohol consumption in pair-bonded male-female pairs of prairie voles (Walcott and Ryabinin, 2017, 2019). The social facilitation of drug intake was observed for alcohol, but not for methamphetamine (Hostetler et al., 2016), highlighting differences in the effects of social environment on actions of these addictive substances.

**Figure 1:**
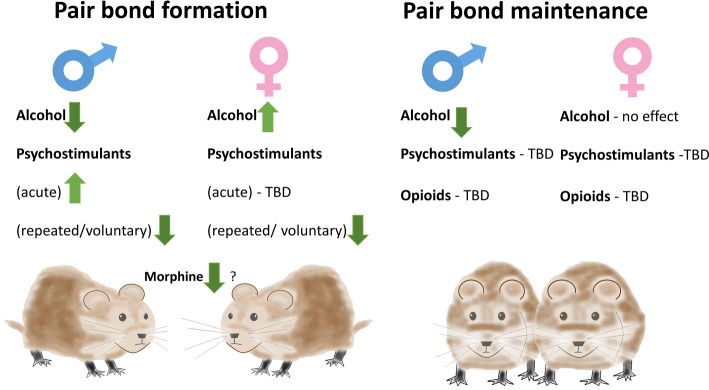
Effects of addictive substances on pair-bonding in prairie voles. Alcohol consumption inhibits pair-bond formation in males but facilitates it in females. Amphetamine administration can either enhance or inhibit pair-bond formation in males depending on timing of administration. Repeated amphetamine administration and methamphetamine drinking inhibit pair-bond formation in both males and females when the exposure occurs 24 h prior to testing partner preference. Morphine can inhibit huddling in male-female pairs. Depending on the partner’s drinking status, alcohol consumption can inhibit pair-bond maintenance in males. Alcohol consumption does not have a significant effect on pair-bond maintenance in females.

While the latter studies highlighted the effects of pair-bond formation on consumption of addictive substances, they did not explain the disruptive effects of substance abuse on social bonds. A different series of studies specifically tested whether such disruptive effects observed in humans could be replicated in prairie voles (Figure 1). An early report demonstrated that administration of morphine attenuated huddling of male-female pairs (Shapiro et al., 1989). This effect was observed with a relatively high dose of morphine (10 mg/kg) also decreasing locomotor activity. The study also did not assess behavior of males and females separately. Nevertheless, it suggested that drugs of abuse can have inhibitory effects on processes indicative of pair-bonding. Subsequent studies showed that injection of AMPH prior to cohabitation could enhance pair-bond formation in male prairie voles and that this effect is dependent on D1R activation (Curtis and Wang, 2007). On the other hand, repeated (three times) AMPH administration in male prairie voles resulted in increased aggression toward female voles, an effect dependent on AVPR1a in the anterior hypothalamus (Gobrogge et al., 2009). Such repeated treatment disrupted formation of PP in male prairie voles. Blocking D1 receptors in the NAcc in this study rescued PP (Liu et al., 2010). Repeated AMPH was also shown to disrupt PP formation in female prairie voles at doses lower than in males, and administration of Oxt into the mPFC restored PP in these females (Young et al., 2014). The apparent contradiction between the first studies showing AMPH inducing PP and the subsequent studies showing inhibition of PP could be due to the fact that in the early study, AMPH was administered acutely and immediately prior to cohabitation, whereas in the subsequent studies, cohabitation happened at least 24 h after the last of repeated injections.

In the studies described above, AMPH was administered by an experimenter. To alleviate the effect of experimenter-induced stress, studies in our laboratory implemented voluntary consumption of drugs to assess their effects on pair-bonding. In these studies, alcohol produced paradoxical sex-dependent effects on pair-bond formation. Alcohol consumption during cohabitation disrupted PP formation in male prairie voles, but facilitated it in females (Anacker et al., 2014a). A number of neural correlates accompanied the differences in PP, including sex-specific changes in the arcuate nucleus, EWcp, MeA, and BNST, suggesting complexity of actions through which alcohol affects pair-bonds. However, their contribution to regulation of pair-bond formation was not causally evaluated. Subsequent experiments mimicked earlier studies on effects of AMPH, but used animals that were voluntarily drinking a solution of methamphetamine during 3 days of cohabitation 24 h before the PP. Similar to the AMPH injection studies, methamphetamine decreased PP formation in both males and females (Hostetler et al., 2016). This effect was accompanied by a decrease in Oxt immunoreactivity in the paraventricular nucleus of hypothalamus (PVN).

There is an obvious difference between most of the above described experiments testing effects of psychostimulants and alcohol on pair-bonding. Alcohol was self-administered just prior to the PP test, whereas in all but one experiment with psychostimulants, there was at least 24 h after the last drug exposure. The alcohol and psychostimulant studies could be comparing acute effects versus effects of withdrawal. Future studies should address this discrepancy. Nevertheless, it is worth noting that one study that tested effects of acute AMPH in male prairie voles found induction of PP (Curtis and Wang, 2007), whereas acute alcohol consumption inhibited PP in male prairie voles (Anacker et al., 2014a), indicating differential effects of these addictive substances on pair-bonding.

The studies above showed that different drugs can have varied effects on the formation of pair-bonds. However, while substance abuse may delay the formation of social bonds, it seems more clinically important to assess its effects on the stability of already established bonds. Moreover, studies in prairie voles indicate that maintenance of the pair-bond requires additional mechanisms beyond those involved in pair-bond formation (e.g., aversion to non-partner animals; Aragona et al., 2006; Resendez and Aragona, 2013). Studies modeling the effects of drugs of abuse on pair-bond maintenance have only been performed recently and only tested the effects of alcohol. These studies show disruption of the established pair-bonds in male prairie voles – as evidenced by decreased PP – when only the male consumes alcohol, but no disruption when both male and female consume alcohol (Walcott and Ryabinin, 2017). Conversely, no disruption of the established pair-bond was seen in females – irrespective of whether the partner consumed alcohol (Walcott and Ryabinin, 2019). Alcohol consumption decreased Oxt in the PVN of males and females regardless of whether bond was disrupted by alcohol or not (Walcott and Ryabinin, 2019). Interestingly, only males demonstrated an increase in immunoreactivity of the activity marker FosB in the periaqueductal gray (PAG) following discordant drinking – suggesting this area may be involved in mediating the effects of discordant drinking on pair-bond maintenance or sensitive to the conditions of discordant drinking (Walcott and Ryabinin, 2017). The PAG is involved in defensive behaviors and romantic love, besides other functions (Depaulis et al., 1992; Acevedo et al., 2012), and needs to be explored in greater detail. We are not aware of studies testing effects of other drugs of abuse on pair-bond maintenance.

The results of these prairie vole studies complement results of the limited epidemiological studies showing that discordant, but not concordant, alcohol consumption is associated with instability of established social bonds. This is important, as the epidemiological studies only assess associations, but not causality of the effects of alcohol. On the other hand, these results also partly contradict epidemiological results in that discordant drinking in the epidemiological studies was associated with instability of social bonds in both males and females. A number of possible explanations for this contradiction have been put forth (Walcott and Ryabinin, 2019). Perhaps most notably, the vole experiments did not assess the same behavior(s) as the human studies on separations; for example, they did not examine actions of the non-intoxicated subject in the PP test. The experimental design of the vole studies contrasts with the epidemiological situation where the initiator of the separation is most likely the low-consuming individual and not the heavy-drinking spouse. Further behavioral data from both preclinical and clinical studies are required to understand the effects of alcohol on pair-bonds; for instance, is the non-intoxicated partner not interacting with the partner consuming the drug, vice versa or mutual?

The involvement of similar neural substrates in pair bonding and addiction has led a number of researchers to suggest that pair-bonding, or even love, is a form of addiction (Insel, 2003; Burkett and Young, 2012). However, we have argued that this similarity could be superficial. Instead, different addictive drugs can “hijack” neurocircuits that are either involved or not involved in various specific social behaviors (Hostetler and Ryabinin, 2012). As a result, different addictive drugs, or even different phases of actions of the same drug (e.g., intoxication versus withdrawal) can have different directions of effects on pair-bonding. Examples of these effects provided in this review (Figure 1) serve as evidence confirming this idea.

Looking forward, what is clearly missing in this literature is a careful examination of effects of different drugs of abuse on maintenance of pair-bonds. So far, only effects of alcohol on this phenomenon have been assessed. Studies on the effects of other drugs of abuse on maintenance of established pair bonds could suggest strategies to help afflicted individuals. Importantly, the prairie vole model is an excellent animal model allowing such future studies.

## Author Contributions

SP performed the literature search, composition, and writing of the manuscript. AR contributed to writing the manuscript.

### Conflict of Interest

The authors declare that the research was conducted in the absence of any commercial or financial relationships that could be construed as a potential conflict of interest.
